# Fabrication of a Hydrophilic Line on a Hydrophobic Surface by Laser Ablation Processing

**DOI:** 10.3390/mi9050208

**Published:** 2018-04-28

**Authors:** Minkyung Kim, Jiwhan Noh

**Affiliations:** 1 Department of Materials Science and Engineering, Korea University, Seoul 02841, Korea; mkyung4188@hanmail.net; 2 Korea Institute of Machinery and Materials (KIMM), 104 Sinseongno, Yuseong-gu, Daejeon 305-343, Korea

**Keywords:** hydrophilic, hydrophobic, nanospikes, microgrooves, laser ablation processing, laser micromachining

## Abstract

A hydrophilic line on a hydrophobic surface was fabricated by using plasma etching and laser ablation processing in this paper. We fabricated the nanospikes on a polyimide surface by using the plasma etching processing. CHF_3_ plasma treatment for hydrophobic coating was conducted on these nanospikes. We fabricated the microgrooves on the hydrophobic nanospikes surface using laser ablation processing. The microgroove had hydrophilic characteristics. In order to measure the hydrophilic characteristics of the microgroove, a solution that was dispersed with silver nanoparticles was used. The hydrophilic line on the hydrophobic surface was dipped in the silver nanoparticle solution. The silver nanoparticles were attached on the hydrophilic microgroove and not on the hydrophobic surface. We concluded that the laser heat affected zone should be reduced for reducing the hydrophilic line width. This hydrophilic pattern on the hydrophobic surface can be used for cell growth, protein manipulation, the spotting of biomolecules, micro-fluidics and water collection. This functional surface can especially be used for an electric flexible circuit, which is newly proposed in this paper.

## 1. Introduction

A hydrophobic surface is a surface that is not friendly to water. It is one of the surfaces that has caught the attention of various industries. Chemical coating is used to create a hydrophobic surface. Generally, fluoride chemicals are coated on the surfaces to make them have a hydrophobic property [1,2]. The weakness of coating with a fluoride chemicals is that the hydrophobic property does not last long. Furthermore, the surface itself must have the hydrophilic property for the fluoride type chemicals to be coated. Therefore, it can be coated on a hydrophilic surface like glass but coating on plastic that has some hydrophobic properties is difficult. As micro/nano fabrication technology advances, there have been studies on superhydrophobic surfaces that use micro/nanopatterns and chemical coating. The difference between superhydrophobic properties and hydrophobic properties is a contact angle. Superhydrophobic properties have a contact angle above 130° and hydrophobic properties have a contact angle between 90° and 130° [3]. Superhydrophobic surfaces have been fabricated by first creating micro/nanopatterns in a shape similar to that of lotus leaves and then adding the chemical coating [4,5,6]. There are also studies on creating a micro pattern on a mold surface and then replicating the micro pattern on a plastic surface for mass production [7]. Because these hydrophobic surfaces have a self-cleaning function, some studies have tried to apply them to areas that clean dirty surfaces [8]. Recently, hydrophobic surfaces that have some parts with a hydrophilic property are being fabricated. There are ongoing studies of applying the hydrophilic patterning function onto hydrophobic surfaces in the areas of cell growth [9,10], protein manipulation [11], the spotting of biomolecules [12,13], micro-fluidics [14,15,16,17] and water collection [18]. In the water collection area particularly, a system for collecting water by naturally mimicking the shell of the Stenocara beetle was developed. On the shell of the Stenocara beetle, hydrophilic lines exist on the hydrophobic surface so that the moisture in the air adheres to the hydrophilic surface and grows larger to become water droplets on the hydrophilic surfaces [19]. As the water droplets grow larger, they flow along the hydrophobic surfaces. The edge of this water flow path is connected to the mouth of the Stenocara beetle so that the Stenocara beetle can drink the water using the moisture in the air. Using the same principle, a system to generate water from the moisture in the air can be produced.

For this study, we fabricated a groove with a width of 15 μm on a hydrophobic surface and have proposed a new hydrophilic patterning method. To fabricate the hydrophobic surface, plasma etching was used to create the nano-sized roughness on a polyimide surface. This surface became hydrophobic through CHF_3_ plasma treatment. Then, a 15 μm wide groove was fabricated by using laser ablation on the hydrophobic surface. This mechanism involves a photo chemical reaction. It represents the material ablation, which occurs after the laser’s photon energy breaks the lattice bonds. When a laser with large photon energy is irradiated on a polymer, the bonds of the polymer lattices are broken down and the material ablates. In other words, the material ablation process begins as the lattice bonding is broken down when photon energy that is larger than the bonding energy of the polymer lattice is irradiated on the polymer [20,21,22,23]. The hydrophilic property of the groove was confirmed by a dipping measurement. To accurately measure the hydrophilic part, a solution that was diffused by silver nanoparticles was used. After immersing the hydrophobic surface with the hydrophilic groove into the silver diffused solution and taking it out, we observed that there were silver nanoparticles left over in the hydrophilic groove. This study indicates that the hydrophilic patterned surface on the hydrophobic surface can be applied to not only cell growth, protein manipulation, the spotting of biomolecules, micro-fluidics and water collection but also to the conductive pattern of a flexible circuit. The reason this study used polyimide as the substrate is because polyimide is widely used as a flexible circuit board and thus the conductive pattern can be used in a flexible circuit. In many papers, research has been conducted on sintering Ag nanoparticles at low temperatures to fabricate flexible electronics [24,25]. However, in this paper, hydrophilic patterns were fabricated by laser ablation on superhydrophobic surfaces and were applied to the conductive pattern of a flexible circuit.

## 2. Experiment Results

### 2.1. Experimental Detail

**Figure 1:**
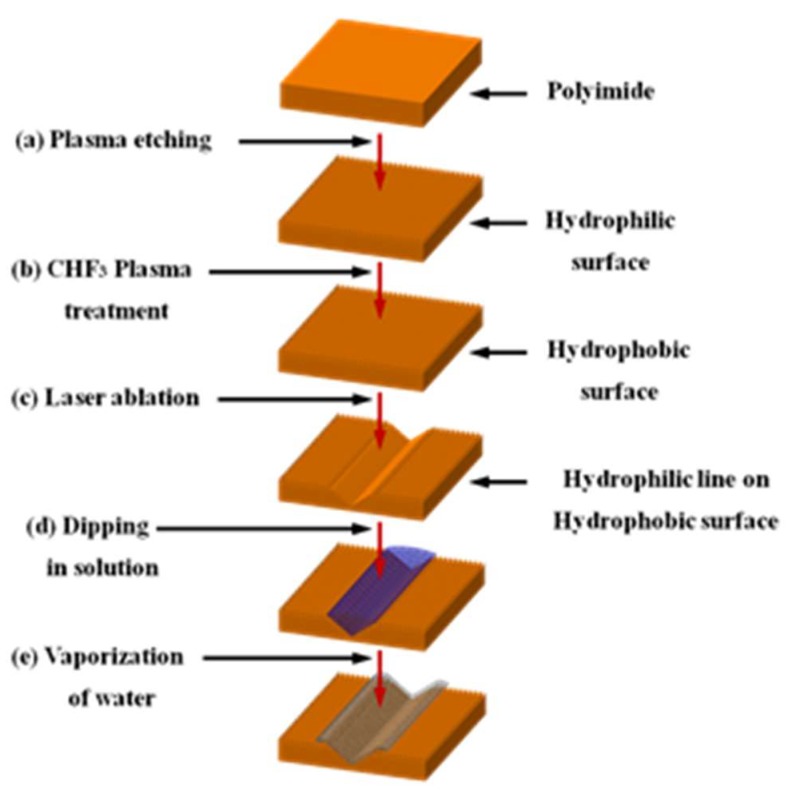
Schematic representation of fabrication processing. (**a**) Nano-sized bumps were formed by plasma etching on the polyimide surface. (**b**) CHF_3_ plasma treatment was performed to make the hydrophobic surface. (**c**) The laser ablation was performed on the hydrophobic surface to make a groove. (**d**) The polyimide was immersed in the silver diffused solution. (**e**) The polyimide was placed in a furnace to evaporate the water in the solution.

Figure 1 shows the overall process of this study. The process shown in Figure 1a is for creating a rough surface. The Polyimide (PI) was maintained at a vacuum level of 2.5 × 10^−2^ Torr and treated with oxygen at 60 sccm at 180–200 Torr and at a power of 750 V and 5.45 mA for 10 min. This process was carried out under Ar-based plasma. As a result, nano sized bumps were created on the PI surface. Then 20 μL of deionized (DI) water was dropped on the surface with the nanobumps and spread at a contact angle of 5° or less. After the nanobumps were created, the vacuum was again maintained at 4 × 10^−2^ Torr or less and the CHF_3_ gas was used at 30 sccm under the process pressure of 910–1000 mTorr and at the power of 470 V and 0.47 mA for 20 min for plasma treatment. As a result, the F-ions were coated on the polyimide surface. When the DI water was dropped again, the nanobump surfaces coated the fluoride compounds and the contact angle became 150° or larger. The polyimide surface became hydrophobic after this plasma treatment.

The process shown in Figure 1c focuses the laser beam on the hydrophobic PI to create the laser ablation on the PI surface. For the laser ablation process, a Nd:YVO4 laser of Hyper Rapid NX 532-25 of COHERENT Inc. (Santa Clara, CA, USA)was used. The laser wavelength was 532 nm, the pulse duration was 12 picoseconds, the average power was 73 mW, the rep rate was 50 kHz and the scan speed was 183 mm/s The laser ablation was proceeded under atmospheric pressure instead of a vacuum. The pulse overlap rate was 75.6% and the pulse energy was 1.46 μJ. Since the focused spot size was 15 μm, the fluence was 0.826 J/cm^2^. For laser focusing and scanning, a Galvano mirror and f-theta lens was used. The Galvano mirror was SS-15 D2 of SCANLAB, GmbH (Puchheim, Germany) by RAYLASE and f-theta lens was S4LFT0101 of Sill Optics GmbH (focal length: 121 mm, Wendelstein, Germany) by SILL Optics. The part irradiated by the laser becomes hydrophilic while the other part remains hydrophobic. If the surface is laser irradiated for a longer time more energy will be deposited on the surface. When the energy exceeds the ablation threshold of the material, the material will begin to be removed. As the material is removed, a V shaped micro groove is created on the material surface. The reason for the V shape is because the focused laser beam has Gaussian distribution.

The process shown in Figure 1d is the step of immersing the polyimide that has both hydrophobic and hydrophilic parts in the silver diffused solution of the PR series (InkTec Co., Asan, Korea). If a surface having the hydrophobic part and hydrophilic part is immersed in normal water and taken out, the water will adhere only to the hydrophilic part. Although the optical microscope is used to observe the wet part, the measurement is not accurate. That is because the water has a 3-dimensional thickness and the image will become blurry when it is out of the depth of focus of the optical microscope. Furthermore, the lighting of the optical microscope often evaporates the water and that degrades the measurement accuracy. As such, this study used the silver diffused solution instead of the water. In this process, the silver diffused solution will adhere only to the hydrophilic part of the polyimide. The surface was then placed in a furnace to evaporate the water in the solution so that only the silver nanoparticles would remain on the hydrophilic surface. To evaporate the water, the sample was put in the furnace at 170 °C for 40 min. After evaporation, the silver nanoparticles on the hydrophilic part were measured using an optical microscope or scanning electron microscope (SEM). If a flexible circuit board was used as the surface, the part with the silver nanoparticles can be used as the conductive circuit after sintering.

### 2.2. Results and Discussion

**Figure 2:**
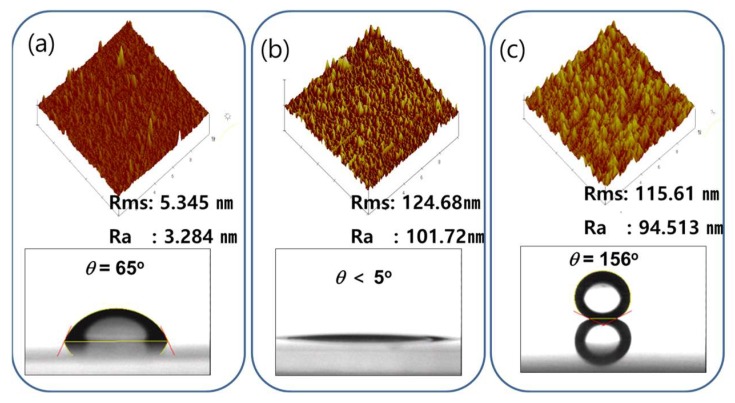
AFM image of nanopatterning using the plasma process and water contact angle. (Root mean square (Rms) means surface roughness value). (**a**) Before the plasma process. (**b**) After the plasma etching process. (**c**) After the CHF_3_ plasma treatment.

Figure 2 shows the Atomic Force Microscope (AFM) image and water contact angle of each step of plasma treatment. Figure 2a shows the AFM image of the original polyimide surface and water contact angle before the plasma treatment. With an average surface roughness (Ra) of 3.2 nm, it has a relatively smooth surface with little roughness. When the surface is passed through a plasma etching process, irregular spike type nanopatterns are created as shown in Figure 2b. The size of the nanopatterns is around 100 nm. Once the nanopatterns are created, the surface will have the hydrophilic property with a water contact angle of around 5°. The water penetrating through the nanopatterns probably causes the low water contact angle. This phenomenon can be explained by the Wenzel State in which water droplets are formed while surrounding all the surfaces of the projections. The smooth surface without the 100 nm patterns had a 65° water contact angle. After the 100 nm patterns are created on the surface, the water contact angle was lowed to 5° as the water penetrated through the nanopatterns. When the surface is treated with CHF_3_ plasma (Figure 2c), the coated part becomes superhydrophobic. In that case, the water cannot penetrate through the nanopatterns and thus a layer of air is created between the nanopatterns and water drops which can be explained by the Cassie-Baxter State. The Cassie-Baxter state is a state in which water droplets are slightly floating on the air or gas layer above the projection [26].

**Figure 3:**
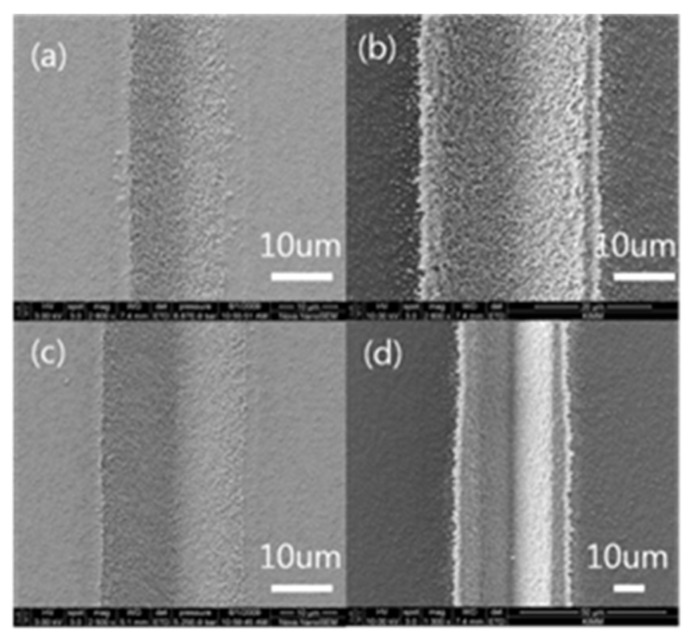

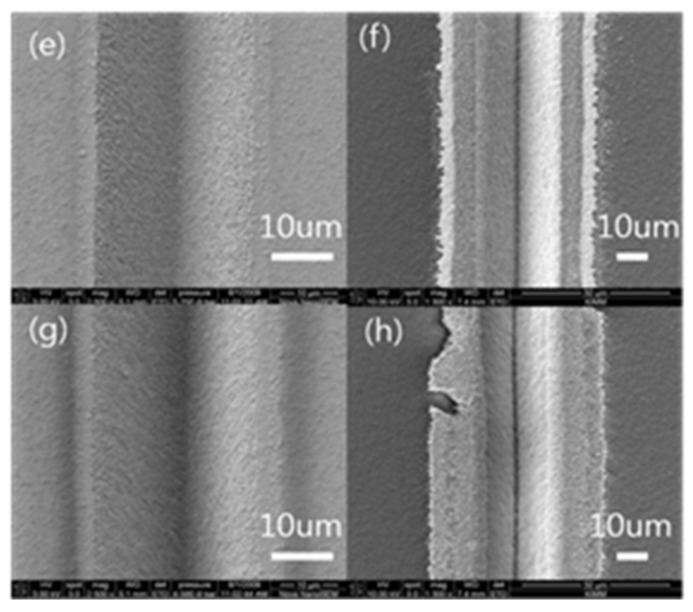
The scanning electron microscopy (SEM) result of laser ablation and sintering. Laser ablation processing (wavelength: 532 nm, pulse duration: 12 picosecond, average power: 73 mW, rep rate: 50 kHz, scan speed: 183 mm/s, (**a**) scan times: 15, (**c**) scan times: 30, (**e**) scan times: 45, (**g**) scan times: 60). (**b**,**d**,**f**) and (**h**) are the dipping and sintering results of (**a**,**c**,**e**) and (**g**) respectively.

Figure 3a,c,e,g show the SEM pictures of the laser treated superhydrophobic surface while Figure 3b,d,f,h show the SEM pictures of the water that evaporated after dipping the laser treated surface into the nanosilver diffused solution. Figure 3a,c,e,g show the SEM pictures of scan times of 15, 30, 45 and 60, respectively. The pictures indicate that the depth of groove increases as the scan time increases. There are many ongoing studies of material ablation by a focused laser beam. When fluence of around 1 × 10^5^ W/cm^2^ is applied to a metallic material, heat is created on the material. If fluence of 1 × 10^5^–1 × 10^6^ W/cm^2^ is applied, the material begins to melt. When fluence of 1 × 10^6^ W/cm^2^ or larger is irradiated on the material, the temperature will exceed the boiling temperature of the material and the material will begin to ablate [27]. Such large fluence can be created by tightly focusing the laser beam. Laser welding, laser cutting and laser drilling use such laser focusing beams. Since laser focusing typically results in a thermal reaction, there will be a thermal effect remaining around the ablated part. When energy is applied to a material, it vibrates the electrons of the material. As the vibration is transmitted to the lattice of the material, the lattices also vibrate. Then this vibration is converted to thermal energy and heat is created in the material. If the energy is large enough, the bonding of the lattices can be broken and the material will begin to ablate. This mechanism was accompanied by a photo chemical reaction. For this study, a 1064 nm laser wavelength was converted to 532 nm using the second harmonic generator in order to induce a photo chemical reaction.

An ultrashort laser was developed as an attempt to reduce the thermal effect part [28,29]. An ultrashort laser is a laser with a pulse duration of several femtoseconds or picoseconds that can be used to minimize the heat-affected zone. When energy is irradiated on a material, the electrons of the material vibrate and the vibration is transmitted to the lattices, which also begin to vibrate. The time that it takes for the energy to be delivered to the lattice is a few picoseconds. The reason the ultrashort laser can reduce the heat affected zone is because the pulse duration is less than that time and thus the laser only generates the electron vibration. As such, this study used a picosecond laser with a pulse duration of 12 picoseconds. To reduce the heat affected zone even further, a laser with a pulse duration in the femtosecond range can be used. But a femtosecond laser is much more expensive than the picosecond laser and offers poor processing time.

Reducing the heat affected zone during laser processing is an important factor of fabricating a small hydrophilic part on a hydrophobic surface. The hydrophilic area will increase if this heat affected zone is large. When the hydrophilic zone, which is patterned on hydrophobic surface, is applied in a flexible circuit, it becomes an electric wire. Therefore, the hydrophilic width must be minimized to reduce the electric wire width and thus integrate as many circuits as possible. To reduce the hydrophilic width, the heat affected zone must be reduced. As shown in Figure 3, more laser irradiation will result in a wider hydrophilic zone, although the width of the micro groove does not increase. Figure 3a shows 15 scan times while Figure 3g shows 60 scan times. As the number of scans increases, a deeper micro groove is then fabricated but then more energy is needed as well. As more energy was irradiated, the heat affected zone increased and the width of the hydrophilic zone also increased. This fact is confirmed by comparison of Figure 3b,h. Figure 3b,h are the result of the surfaces shown in Figure 3a,g, respectively, being immersed in the silver nanoparticle diffused solution and then dried. One can see that in Figure 3h, it shows a wider section of silver nanoparticles than in Figure 3b. To reduce the hydrophilic width, the heat affected zone must be smaller and the number of scans must be reduced. However, if the number of scans is reduced, the groove depth will also decrease. In the application of the hydrophobic and hydrophilic surface on the flexible circuit, less groove depth can be a problem. That is because a deeper groove means a larger space to hold the conductive particles. Having more space to hold the conductive particles means the electric or electronic resistance can be reduced by that much. There are many ongoing studies of reducing the electric wire widths on the flexible circuit. They increase the wire thickness to maintain electric resistance while reducing the wire width.

**Figure 4:**
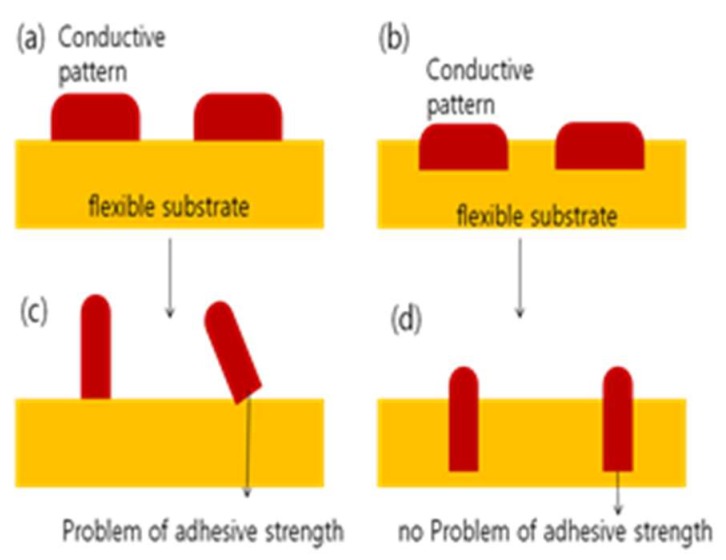
Schematic drawing of a conductive pattern application. (**a**) the case of conductive patterns on flexible circuits. (**c**) the case of reducing the wire width while maintaining electrical resistance in (**a**). (**b**) the case of conductive patterns on flexible circuits with the micro groove. (**d**) the case of reducing the wire width while maintaining electrical resistance in (**b**).

If the wire thickness is increased and the width is decreased, the possibility of the wire being peeled off from the flexible substrate will increase. Figure 4a,c show the cases of conductive patterns on flexible circuits. To reduce the wire width while maintaining the electric resistance, the height of the conductive pattern must increase as shown in Figure 4c. However, the conductive pattern will be more likely to be peeled off from the flexible substrate in that case. To prevent this, a micro groove may be fabricated on the flexible substrate and the conductive particles should be put in substrate as shown in Figure 4b,d. Figure 4b shows the case of a wide conductive pattern and Figure 4d shows the case of a narrow conductive pattern. If the electric wire width must be reduced as shown in Figure 4d, the groove depth may be increased to expand the space to pack the conductive material and thus enable reduction of the wire’s width while maintaining electrical resistance. That way, the problem of the conductive pattern being peeled off, as shown in Figure 4c, can be resolved. Since the circuits are usually bent in the flexible circuit, this peeling-off problem has become a key issue.

However, there is a limitation to how deeply the groove can be fabricated. In the process of fabricating the hydrophilic pattern by using the laser on the hydrophobic surface proposed in this paper, the heat affected zone will increase as the depth of the micro groove increases. A larger heat affected zone will widen the hydrophilic part and it will eventually increase the width of the electric wire. As shown in Figure 3h, increasing laser irradiation to increase the micro groove depth will expand the heat affected zone and it also increases the hydrophilic width. This also will eventually mean that the electric wire width will increase. Therefore, the number of laser scans and laser power will need to be determined according to the required electric resistance and electric wire width.

To reduce the width of the hydrophilic part on the hydrophobic surface, the laser-focused spot may be reduced in addition to reducing the heat affected zone. To reduce the laser spot, the laser wavelength should be reduced or the focal length of the focus lens should be made smaller. Otherwise the larger beam of the laser irradiated focus lens will result in a smaller focused spot. However, if the laser-focused spot size is reduced, the depth of focus, which is the zone in which the focused spot size is maintained at a constant, will also be decreased. When this depth of focus is decreased, an auto focus system to maintain the certain distance between the laser focus lens and material will be needed during the laser process. The auto focus system is particularly needed on a flexible surface since it is difficult to maintain a specific height on a flexible surface. However, it is very difficult to use auto focusing while maintaining the scan speed of 183 mm/s, which was used in this study. Therefore, this study adopted a laser spot size of around 15 μm for laser processing. This enabled the micro groove of a specific width without having to use the auto focus system.

**Figure 5:**
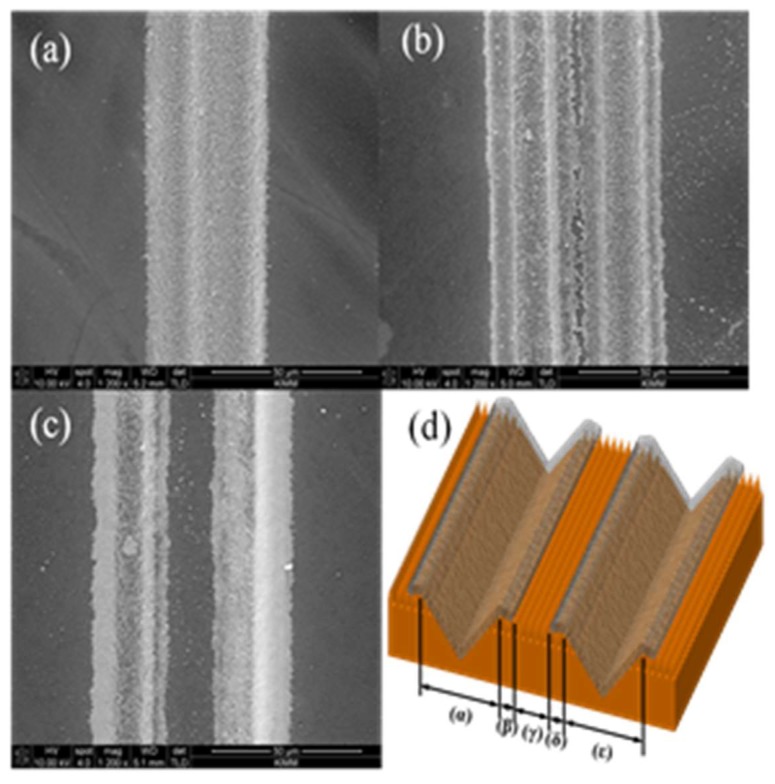
The SEM image of adjacent hydrophilic lines (**a**) Line interval: 20 μm, (**b**) Line interval: 30 μm, (**c**) Line interval: 40 μm, (wavelength: 532 nm, pulse duration: 12 picoseconds, average power: 73 mW, rep rate: 50 kHz, scan speed: 183 mm/s, scan times: 15) (**d**) Schematic representation of adjacent hydrophilic lines.

Another important factor, in addition to the peeling-off problem, of using the hydrophilic pattern on a hydrophobic surface in a flexible circuit is how close two conductive lines can be laid out next to each other. It is very important to place two conductive lines as close to each other as possible in the case of a transistor since the gap between the source conductive line and drain conductive lines determines the performance of the transistor. Figure 5 shows how close two hydrophilic lines can be placed together. The laser wavelength was 532 nm, the average power was 73 mW, the rep rate was 50 kHz, the scan speed was 183 mm/s and the number of scans was 15. Figure 5a–c show the gaps between the two lines of 20 μm, 30 μm and 40 μm, respectively. SEM pictures were taken after immersing the laser treated hydrophilic lines that were fabricated on a hydrophobic surface into the nanoconductive particle diffused solution and drying them. Since the hydrophilic line width was 15 μm, there was no hydrophobic area between the two lines when the line gap was 20 μm or 30 μm and thus the conductive particles completely covered up all the areas. However, when the gap between two hydrophilic lines was 40 μm, there was a hydrophobic area between the lines and no conductive particles were on that hydrophobic area. Figure 5d shows the schematic representation of adjacent hydrophilic lines. In Figure 5d, (α) and (ε) show the fabricated hydrophilic lines, (β) shows the heat affected zone that was created when the hydrophilic line (α) was fabricated, while (δ) shows the heat affected zone created when the hydrophilic line (ε) was fabricated. (γ) shows the hydrophobic area existing between two hydrophilic lines. In the case of the conductive patterns, if (α) and (ε) were processed deeply in order to reduce electrical resistance, the created heat affected zones (β) and (δ) will increase. As a result, the (γ) area is likely to be eliminated and two conductive lines may overlap. In that case, the gap between (α) and (ε) must be widened. However, it must be noted that in that case, the transistor’s performance will deteriorate. To place the two hydrophilic lines closer in order to improve the transistor performance, the heat affected zone must be reduced. To reduce the heat affected zone, the depth of the hydrophilic groove must be decreased and that will result in higher electric resistance. Therefore, the groove depth and gap between two hydrophilic lines must be determined after considering all these conditions. The heat affected zone will be influenced by the depth of the groove fabricated by the laser processing and that influence will also affect how close the two conductive lines can be placed.

## 3. Conclusions

This study fabricated a groove by using laser processing on a hydrophobic surface. When this surface is immersed into a solution and taken out, the solution will remain only in the groove, since the groove has a hydrophilic property. To accurately measure the hydrophilic part, a silver nanoparticle diffused solution was used in this study. After evaporating the water in the groove, the residual silver nanoparticles were observed to measure the hydrophilic part. The measurement indicates that the hydrophilic width increased as the heat affected zone increased. The fabricated hydrophilic pattern on a hydrophobic surface described in this paper can be used for cell growth, protein manipulation, the spotting of biomolecules, micro-fluidics, water collection and conductive patterns on a flexible circuit.
